# Respiratory and GIT tract immune responses of broiler chickens following experimental infection with Newcastle disease’s virus

**DOI:** 10.1007/s00580-018-2728-z

**Published:** 2018-05-09

**Authors:** Hadi Rohollahzadeh, Hassan Nili, Keramat Asasi, Saeed Mokhayeri, Amir Hossein Asl Najjari

**Affiliations:** 1 Resident of Poultry Science, School of Veterinary Medicine, University of Shiraz, Shiraz, Iran; 2 Isfahan Univeristy, Isfahan, Iran; 3 Department of Clinical Studies, School of Veterinary Medicine, University of Shiraz, Shiraz, Iran

**Keywords:** Newcastle disease, Cellular immunity, T lymphocyte, Immunohystochemistry, Broiler

## Abstract

Newcastle disease causes a lymphoproliferative response in the tracheal and intestinal mucosa of the infected birds. In this study, the Hitchner B1 and I-2 vaccine and challenging of ND field strains were used to evaluate the populations of T lymphocyte subsets infiltrated intestinal and tracheal, also to shed some light on cell-mediated immune response using enzyme-linked immunosorbent assay (ELISA) detecting chicken’s serum interferon-γ. Three hundred-day-old broilers were randomly divided into four groups. Groups 1 and 2 received I-2 and B1 vaccines, respectively, while groups 3 and 4 were challenged-unvaccinated and unchallenged-unvaccinated groups. Blood samples were taken from five random chicks and were then tested with ELISA test. Three chicks of each group were euthanized after vaccine administration and also challenging with acute virus. Interferon-γ changes were significant in time (*p* < 0.001). Totally, there was no significant difference between I-2 and B1 groups. The number of CD3+, CD4+, and CD8+ cells of I-2 and B1 vaccinated group’s intestine and the trachea samples was significantly increased compared with the negative control group (*p* < 0.001). The results indicated the significant increase in CD4+ and CD8+ in intestinal and tracheal tissues, while the level of interferon-γ of the vaccinated group was more than the unvaccinated one. Finding no significant differences between the vaccinated groups indicated the potential of both vaccines in producing CD4+ and CD8+ in the tracheal and intestinal tissues and the equality of interferon-γ production in the sera.

## Introduction

Newcastle disease is one the most contagious viral diseases which could infect almost all species around the world. Vaccination is a practical and useful way against the endemic cases of Newcastle disease (Usman 2002). Currently, common vaccination programs against Newcastle disease in most countries include administering live attenuated and killed vaccines to control the endemic strains. Vaccines belonging to B1 and LaSota strains are the commonest vaccines used to prevent Newcastle disease in the poultry industry though sometimes facing with vaccine failures due to unknown reasons. I-2 vaccine against Newcastle disease is becoming more popular not only in village chicken but also in industrial broiler’s one. This study was conducted to elaborate some aspects of cell-mediated immunity of this vaccine compared to a commonly used commercial vaccine against Newcastle disease. Several studies proved I-2 vaccine as an appropriate choice with good immunogenicity, low level of vaccine reaction, less expenses with vaccine production in comparison with the common vaccines, and thermostability making the vaccine no need to follow cold chain during the transportation (Bell 2001). Fifty percent embryo infectious dose (EID50) of the virus used for the vaccine was supposed to be 10^6/5^ since EID50/bird higher 10^6^ was reported as the immunizing titer in the studies conducted on the protective antibody titers against I-2 virus by other researchers (Nili et al. 2005). If the vaccine was kept in 4–8 °C for a long time, at least a year, it could result in achieving an acceptable antibody titer. I-2 vaccine could be administered via different routes including eye drop, drinking water, feed, and injection. However, most farmers prefer to administer the vaccine through the eye drop route (Bensink and Spradbrow 1999).

The first line of specific immune response to NDV is cellular immunity appearing in 2–3 days after administering ND live vaccines. It is common to assess the effects of Newcastle vaccines with specific antibody titers against the vaccine. Titration of the sera after administrating the vaccines indicates the outcome of the vaccine and whether the resulted titer results in the immunization or not (OIE 2012; Swayne et al. 2013). Ghumman et al. used leukocyte migration inhibition assay to evaluate the cellular immunity in 1976 and indicated that lymphocyte mitogens were occurred 2 days after the primary vaccination. This finding was related to bird’s resistance against the challenged virus. They concluded that the cellular immunity against certain strains of NDV is an integral part of the total immunity together with other immunity parameters such as the humoral and the local (Ghumman and Bankowski 1976; White and Appleton 1953). It was also indicated that although bursectomized chicks received Newcastle vaccines were incapable of producing antibodies up to immunization level, they were resistant to the disease. In addition, Newcastle disease HI titer was not depended on cellular immunity response (Marino and Hanson 1987). However, since the role of immunity is clear in all poultry diseases, it is necessary to study the immunity system more to develop and modify the prevention strategies (Lillehoj and Trout 1993). Cellular immunity is a type of specific adaptive immunity with T lymphocytes and important roles in developing the immunity in vaccinated chicks against the Newcastle disease virus and eliminating it (Sharma 1999). T cells are classified based on the cellular evolution and expression to CD4 and CD8 co-receptors. CD8 is usually expressed on the surface of cytotoxic T cells, while CD4 is found on the surface of T helper cells (Chan et al. 1988). Glycoprotein antigens on the surface of leukocytes are biomarkers or cluster of differentiation (CD) that could be used to differentiate the leukocytes based on the cell type and the maturity stage (Saalmuller et al. 1997; Glick 2000). Also, several studies indicated that measuring T cell-released interferon-γ after in vitro or in vivo stimulation could be also an appropriate marker to evaluate the cellular immunity after the infection or vaccination (Breed et al. 1997; Karaca et al. 1996; Martin et al. 1994; Prowse and Pallister 1989). It became obvious that the chicken’s body also uses several mechanisms in addition to producing Newcastle disease virus specific antibody against the virus (Marino and Hanson 1987). Enzyme-linked immunosorbent assay (ELISA) indicated some reliable and simple methods to evaluate the cytokine release after the activation proving this parameter as an appropriate one to assess the cellular immunity (Mateu de et al. 1998; Whiteside 1994; Rothel et al. 1990; Mateu de et al. 1998). Several cell-mediated immunity (CMI)-associated cytokines have been detected in chickens during the recent years giving the chance to develop a new immunologic assay for the species. Recently, monoclonal antibodies (mAbs) were developed based on specific interferon-γ ELISA test (Lambrecht et al. 2000). The aim of this study was to evaluate the cellular immunity with serum interferon-γ using ELISA assay, to study the changes in T cell subsets, CD4 and CD8, in intestinal and tracheal samples with monoclonal antibodies against the surface or intracellular glycoproteins as the markers using immunohistochemistry assay after vaccinating with I-2 thermostable vaccine and to compare the results of this vaccine with acute virus and B1 vaccine challenges.

## Materials and methods

### Vaccines and antigens

B1 lentogenic live vaccine (Razi Vaccine and Serum Research Institute, Karaj, Iran) and I-2 thermostable vaccine (Isolate Department, School of Veterinary Medicine, Shiraz University, Iran) were used in this study.

## Characteristics of the challenging virus

Herts 33.56 (ICPI 1.88) was used in this study as an international acute strain. This virus was originally isolated from Hertforshir chicken in England in 1933 (Allan et al. 1978). Herts 33.56 was propagated in 9-day-old to 11-day-old embryonated chicken eggs. Concentration of the virus in infected chick embryo allantoic fluid was 10^8^ EID50/mL. As the dose of challenge virus, determined in previous studies, 1 × 10^−4^ EID50/mL was the basic dose of the challenge in this study (Alexander et al. 2006).

### Experiment plan

To compare the efficacy of I-2 thermostable with B1 Newcastle disease vaccines, 300-day-old Cobb broiler chicks were divided into four equal groups. The first vaccine of vaccinating group’s chicks was administered on day 19 through eye drop route, while the booster vaccine was also administered 7 days later, on 26-day-old chicks, by the same route. Fourteen days after second vaccine administration on 40-day-old chicks, all groups except group 4 as the control one were challenged with Herts 33.1956 with EID50:10^4^ through the nostril route) Table 1).

**Table 1:** Vaccination of groups 1 and 2 using I-2 and B1 vaccines, respectively, followed by challenging with Herts 33.56 (ICPI 1.88). Groups 3 and 4 were also positive and negative control groups, respectively

Group	Vaccine	First vaccination	Booster vaccination	Challenge day
1	I-2	19-day-old	26-day-old	40-day-old
2	B1	19-day-old	26-day-old	40-day-old
3	–	–	–	40-day-old
4	–	–	–	–

### Measurement of chicken IFN-γ in the collected serum

Blood samples were taken from five random chicks 3 and 7 days after the first vaccine administration and also 3, 7, and 14 days after the second vaccine administration and challenging with acute NDV to measure interferon-γ by a solid-phase sandwich-ELISA (Chicken IFN-γ Commercial ELISA test kit, Shanghai Crystal Day Biotech Co., Ltd.).

### Assessing T cell changes with immunohistochemistry method

Three chicks of each group were euthanized randomly 7 days after the primary vaccine administration, 3, 7, and 14 days after second vaccine administration, and 7 days after challenging with acute virus to take the medial part of the trachea and the medial part of duodenum and to assess the changes in T cell subsets, CD4 and CD8, resulted from administering various vaccines using immunohistochemistry method. Four to 5-μm paraffin-embedded sections were prepared following fixation in 10% neutral buffered formalin (Merck, Darmstadt, Germany) for 18–24 h. Briefly, sections were heated at 60–63 °C for 15 min and incubated with xylene and 100 and 96% ethanol for IHC de-paraffinization and rehydrating. Then, sections were incubated with hydrogen peroxide/methanol solution to remove endogenous peroxidise activity at room temperature. Sections were treated with Tris-EDTA (buffer pH = 9) in 90–93 °C for 20 min as the heat-induced epitope retrieval step while using goat serum as a blocking solution in PBS. Thereafter, sections were incubated with the murine anti-CD4, CD8, and CD3 mAbs (Lifespan Bioscience, USA) at a dilution of 1:100 for 2 h in 37 °C. Sections were washed with phosphate-buffered saline (PBS) buffer 1× and incubated with Dako ENVISIN™ kit (Dako, Carpinteria, CA, USA) for 25 min at room temperature and were eventually stained with 3,3-diaminobenzidine (DAB; Sigma, Aldrich). All cells were counted using CD4+, CD8+, and CD3+ markers in five random microscope fields (×400 magnification) by a single person. The average of positive cells in ×400 magnification of the microscope was calculated for each cell and sample (Awad et al. 2014). Control staining was also performed to detect any possible immunity reactions to PBS instead of primary (mAb). No stain was observed in the control group.

### Statistical analysis

The statistical analysis of the study was performed with the 95% level of significance using SPSS software (IBM. USA) version 23. One-way analysis of variance (ANOVA) method was also used to compare the groups, while the least significant difference was also considered. All data were significant at the level of *p* < 0.05.

## Results

### Studying the clinical signs of various groups and different days

Before the challenge, none of the groups showed any clinical and necropsy signs. Three days after the challenge, three cases of paralysis were noticed in group 2, but no clinical cases of the disease were observed in group 1. In the positive control group, lethargy and anorexia together with some other signs such as head inflammation and ruffled feathers were noticed 2–3 days after the challenge. Since the fourth day after the challenge, the mortality onset was noticed with some signs including green and watery diarrhea, respiratory signs, head tremors, and torticollis. The mortality trend was severe, and 6 days after the challenge, all birds of the group were dead**.**

## Interferon-γ levels of test groups

**Fig. 1:**
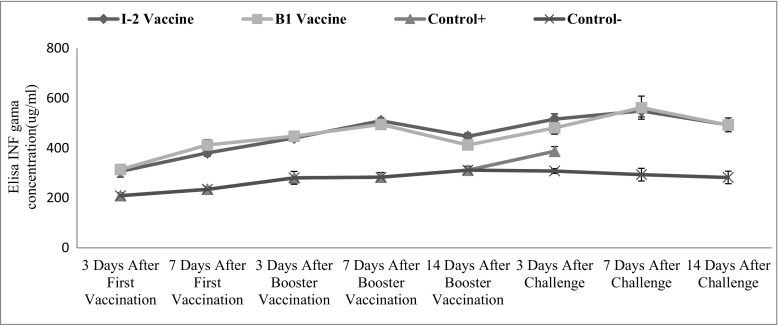
Changes of INF-γ production in different groups and time following vaccination with I-2/B1 and challenge with ND virus

Interferon-γ changes were significant in time (*p* < 0.001). Totally, there was no significant difference between I-2 and B1 groups, but 7 days after primary vaccination and 14 days after the booster, a significant difference was observed. The statistical difference was significant between I-2 group and negative control group, and finally, the differences of this parameter between various groups and on different days were significant (*p* < 0.001) (Table 2) (Fig. 1).

**Table 2:** The level of interferon gamma production (pg/mL) in the bird’s serum in response to the NDV vaccination and challenge

First vaccination			Booster vaccine			After challenge		
Groups	3DPV	7DPV	3DPV	7DPV	14DPV	3DPC	7DPC	14DPC
I-2 vaccine	306.05 ± 22.3a	380.31 ± 12.8a	440.1 ± 9.92a	509.26 ± 12.6a	446.5 ± 13.5a	516.15 ± 21.1a	548.73 ± 24.2a	492.6 ± 27.8a
B1 vaccine	314 ± 19.2a	412.02 ± 22a	447.18 ± 8.39a	494.86 ± 10.1a	412.44 ± 9.22a	480.81 ± 25.1a	561.88 ± 46.3a	492.1 ± 10.8a
Unvaccinated-challenged	208.68 ± 11.4b	234.31 ± 11.04b	280.28 ± 25.23b	283.06 ± 18.7b	311.12 ± 15.8b	386.84 ± 19.1b	–	–
Unvaccinated-unchallenged	208.68 ± 11.4b	234.31 ± 11.04b	280.28 ± 25.23b	283.06 ± 18.7b	311.12 ± 15.8b	307.67 ± 9.67b	293.31 ± 25b	281.97 ± 25b

Different letters shows statistical significance (*p* ≤ 0.001) between groups in each column

*DPV* day post-vaccination, *DPC* day post-challenge

### Assessing T cell community and total lymphocyte changes (CD4+, CD8+) with immunohistochemistry

To do so, tracheal and intestinal samples of Newcastle-infected broilers were stained with immunohistochemical lymphocyte markers and then studied according to the distribution of various subsets of the lymphocytes (Tables 3, 4, 5, and 6).

**Table 3:** Changes in T lymphocyte subpopulations expressing CD3+ in the trachea and intestine post-infection with NDV

Days after first vaccination			Days after booster vaccination						Days after challenge	
7DPV			3DPV		7DPV		14DPV		7DPC	
Groups	Trachea	Intestine	Trachea	Intestine	Trachea	Intestine	Trachea	Intestine	Trachea	Intestine
I-2 vaccine	15 ± 2a	50.67 ± 5.1a	23 ± 4.3a	55 ± 6.2a	25 ± 3a	50 ± 2.6a	22 ± 2.6a	43 ± 4a	29 ± 3.4a	50 ± 1.7a
B1 vaccine	14 ± 3.05a	46.33 ± 5.1a	26 ± 3a	48 ± 6a	27 ± 3.6a	48 ± 3.6a	26 ± 1.7b	42.67 ± 3.5a	25 ± 1.7a	48 ± 3.4a
Unvaccinated-challenged	6.67 ± 1.1b	19 ± 2b	9 ± 1.7b	16 ± 3.6b	9 ± 1.7b	9 ± 2b	9 ± 1c	11 ± 1b	–	–
Unvaccinated-unchallenged	6.67 ± 1.1b	19 ± 2b	9 ± 1.7b	16 ± 3.6b	16 ± 3.6b	9 ± 2b	9 ± 1c	11 ± 1b	7 ± 1b	13 ± 3b

Different letters shows statistical significance (*p* ≤ 0.001) between groups in each column

*DPV* day post-vaccination, *DPC* day post-challenge

**Table 4:** Changes in T lymphocyte subpopulations expressing CD4+ in the trachea and intestine post-infection with NDV

Days after first vaccination		Days after booster vaccination							Days after challenge	
7DPV		3DPV			7DPV		14DPV		7DPC	
Groups	Trachea	Intestine	Trachea	Intestine	Trachea	Intestine	Trachea	Intestine	Trachea	Intestine
I-2 vaccine	14 ± 3a	48 ± 8.1a	25 ± 3a	54 ± 3.6a	33 ± 4.3a	62 ± 4a	29 ± 2.6a	55 ± 4a	33 ± 2.6a	59 ± 2.6a
B1 vaccine	17 ± 3a	44 ± 4a	28.7 ± 2.3a	50 ± 3.4a	33 ± 6a	57 ± 1.7b	22.3 ± 1.1b	50.3 ± 3.2a	29 ± 3a	58.67 ± 3.7a
Unvaccinated-challenged	9.3 ± 1.5b	20.3 ± 1.5b	9 ± 2b	18.6 ± 1.5b	12 ± 3.4b	15 ± 1c	15 ± 1.7c	15 ± 2b	–	–
Unvaccinated-unchallenged	9.3 ± 1.5b	20.3 ± 1.5b	9 ± 2b	18.6 ± 1.5b	12 ± 3.4b	15 ± 1c	15 ± 1.7c	15 ± 2b	8 ± 1.7b	19 ± 2.6b

Different superscripts shows statistical significance (*p* ≤ 0.001) between groups in each column

*DPV* day post-vaccination, *DPC* day post-challenge

**Table 5:** Changes in T lymphocyte subpopulations expressing CD8+ in the trachea and intestine post-infection with NDV

Days after first vaccination			Days after booster vaccination						Days after challenge	
7DPV			3DPV		7DPV		14DPV		7DPC	
Groups	Trachea	Intestine	Trachea	Intestine	Trachea	Intestine	Trachea	Intestine	Trachea	Intestine
I-2 vaccine	12.3 ± 2.5a	43 ± 7a	22.3 ± 4.6a	61 ± 4.3a	37 ± 3.4a	66 ± 2.6a	32.6 ± 1.5a	62 ± 2.6a	37 ± 2.6a	66 ± 5.5a
B1 vaccine	15.6 ± 2a	40 ± 2.6a	28.6 ± 1.5b	59 ± 6.5a	38 ± 2a	69 ± 1.7a	29 ± 2b	63 ± 6a	34 ± 2.6a	68 ± 2.6a
Unvaccinated-challenged	5.3 ± 1.5b	11 ± 2b	5.33 ± 1.5c	11 ± 2.6b	5 ± 2b	9 ± 1.7b	5 ± 2c	9 ± 1.7b	–	–
Unvaccinated-unchallenged	5.3 ± 1.5b	11 ± 2b	5.33 ± 1.5c	11 ± 2.6b	5 ± 2b	9 ± 1.7b	5 ± 2c	9 ± 1.7b	5 ± 1.7b	11 ± 2b

Different superscripts shows statistical significance (*p* ≤ 0.001) between groups in each column

*DPV* day post-vaccination, *DPC* day post-challenge

**Table 6:** Changes in T lymphocyte subpopulations expressing CD4+/CD8+ in the trachea and intestine post-infection with NDV

Days after first vaccination			Days after booster vaccination						Days after challenge	
7DPV			3DPV		7DPV		14DPV		7DPC	
Groups	Trachea	Intestine	Trachea	Intestine	Trachea	Intestine	Trachea	Intestine	Trachea	Intestine
I-2 vaccine	1.13a	1.11a	1.15a	0.88a	0.89a	0.94a	0.88a	0.89a	0.89a	0.89a
B1 vaccine	1.08a	1.10a	0.99a	0.85a	0.88a	0.82a	0.77a	0.80a	0.85a	0.88a
Unvaccinated-challenged	1.82b	1.88b	1.74b	1.78b	2.65b	1.7b	2.58b	1.72b	–	–
Unvaccinated-unchallenged	1.82b	1.88b	1.74b	1.78b	2.65b	1.7b	2.58b	1.72b	1.72b	1.76b

Different superscripts shows statistical significance (*p* ≤ 0.001) between groups in each column

*DPV* day post-vaccination, *DPC* day post-challenge

**Fig. 2:**
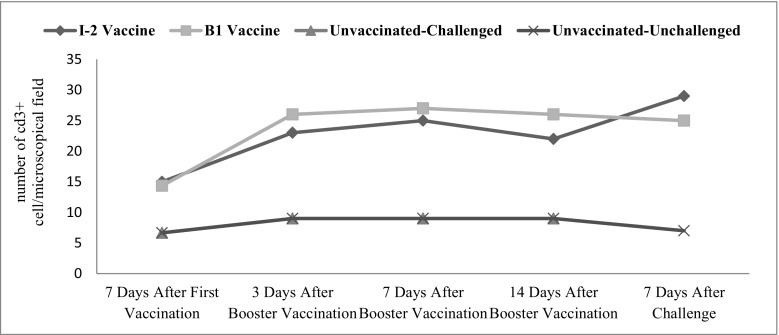
Subpopulations of tracheal CD3+ changes following vaccination with I-2/B1 and challenge with ND virus in different groups and time

Tracheal CD3+ changes were significant in time (*p* ˂ 0.001). The vaccination route and vaccine type also significantly affected the level of this factor in the tracheal (*p* ˂ 0.001) (Fig. 2).

**Fig. 3:**
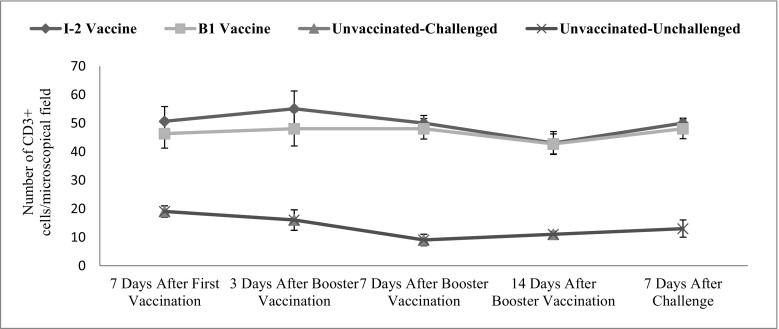
Subpopulations of intestinal CD3+ changes following vaccination with I-2/B1 and challenge with ND in different groups and time

Intestinal CD3+ changes were significant in time (*p* = 0.008). The vaccination route and vaccine type also significantly affected the level of this factor in the intestine (*p* ˂ 0.001) (Fig. 3).

**Fig. 4:**
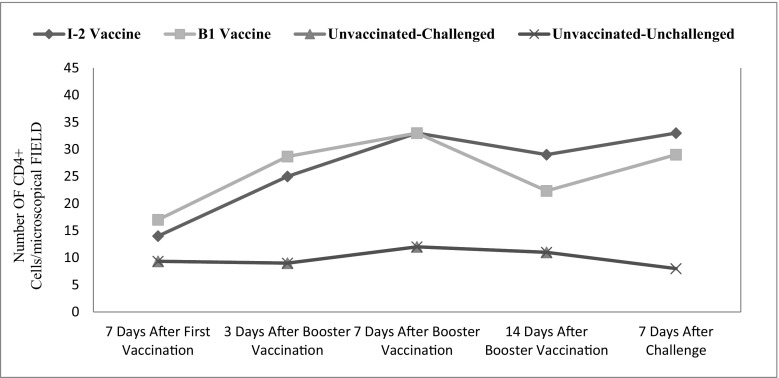
Subpopulations of tracheal CD4+ changes following vaccination with I-2/B1 and challenge with ND virus in different groups and time

Tracheal CD4+ changes were significant in time (*p* ˂ 0.001). The vaccination route and vaccine type also significantly affected the level of this factor in the trachea (*p* ˂ 0.001) (Fig. 4).

**Fig. 5:**
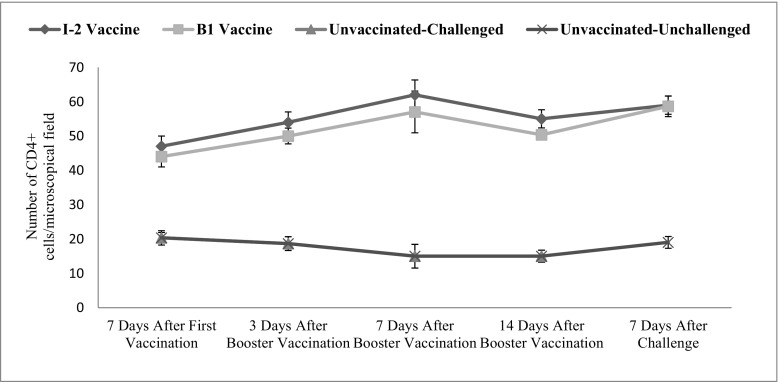
Subpopulations of intestinal CD4+ changes following infection with NDV in different groups and time

Intestinal CD4+ changes were significant in time (*p* = 0.031). The vaccination route and vaccine type also significantly affected the level of this factor in the intestine (*p* ˂ 0.001) (Fig. 5).

**Fig. 6:**
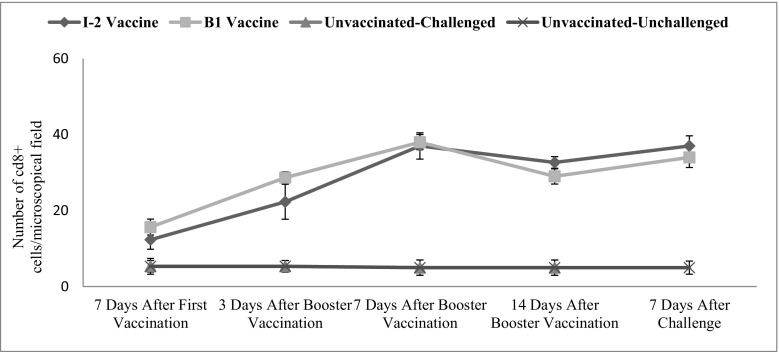
Subpopulations of tracheal CD8+ changes following vaccination with I-2/B1 and challenge with ND virus in different groups and time

Tracheal CD8+ changes were significant in time (*p* ˂ 0.001). The vaccination route and vaccine type also significantly affected the level of this factor in the tracheal (*p* ˂ 0.001) (Fig. 6).

**Fig. 7:**
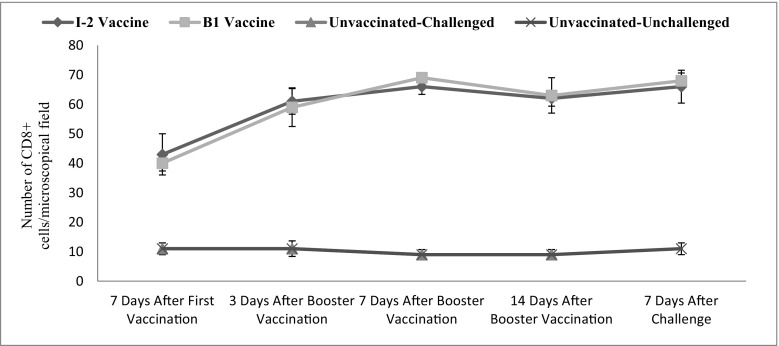
Subpopulations of intestinal CD8+ changes following vaccination with I-2/B1 and challenge with ND virus in different groups and time

Intestinal CD8+ changes were significant in time (*p* ˂ 0.001). The vaccination route and vaccine type also significantly affected the level of this factor in the intestine (*p* ˂ 0.001) (Fig. 7).

**Fig. 8:**
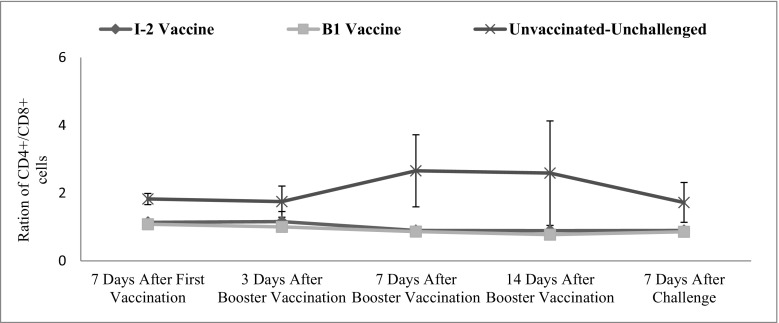
Subpopulations of tracheal CD4+/CD8+ changes following vaccination with I-2/B1 and challenge with ND virus in different groups and time

Tracheal CD4+/CD8+ changes were not significant in time. In other words, there was no significant difference between the average of this ratio in various time points (*p* = 0.219). The vaccination route and vaccine type also significantly affected the level of this factor in the trachea (*p* = 0.010) (Fig. 8).

**Fig. 9:**
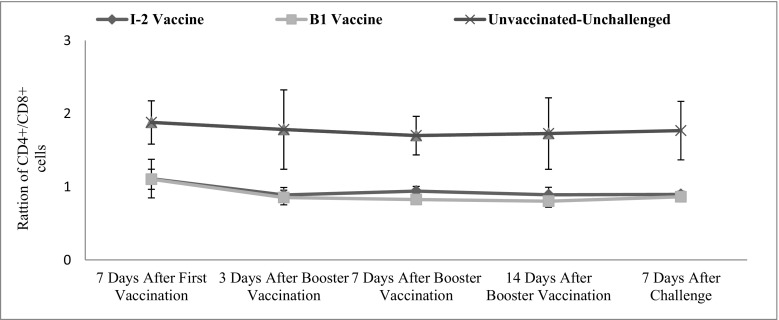
Subpopulations of intestinal CD4+/CD8+ changes following vaccination with I-2/B1 and challenge with ND virus in different groups and time

Intestinal CD4+/CD8+ changes were not significant in time. In other words, there was no significant difference between the average of this ratio in various time points (*p* = 0.402). The vaccination route and vaccine type also significantly affected the level of this factor in the intestinal (*p* ˂ 0.001) (Fig. 9).

**Fig. 10:**
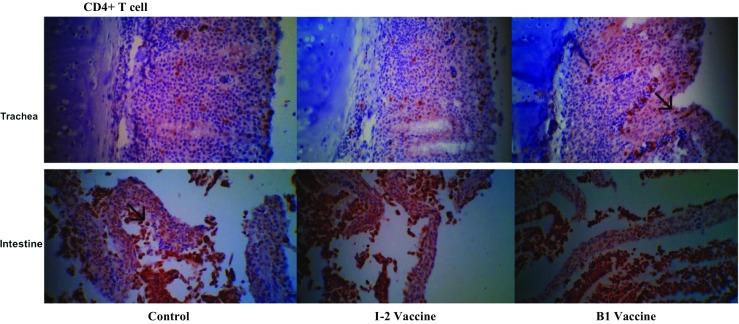
Immunohistochemistry representative images of tracheal and duodenal samples of the I-2 thermostable and B1 vaccinated groups and the unvaccinated control group. Stained with CD4+ monoclonal antibody and sectioned 3 days post-booster vaccine administration. ×400 magnification

**Fig. 11:**
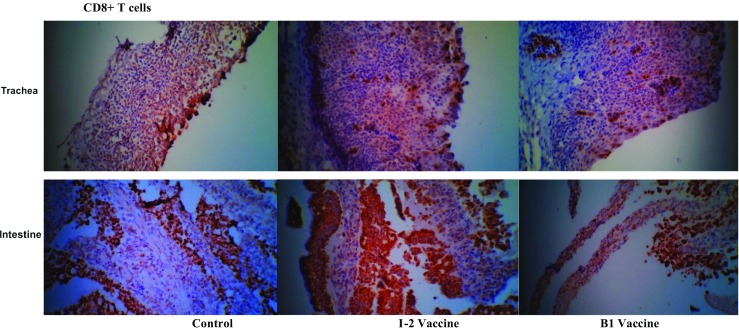
Immunohistochemistry representative images of tracheal and duodenal samples of the I-2 thermostable and B1 vaccinated groups and the unvaccinated control group. Stained with CD8+ monoclonal antibody and sectioned 3 days post-booster vaccine administration. ×400 magnification

**Fig. 12:**
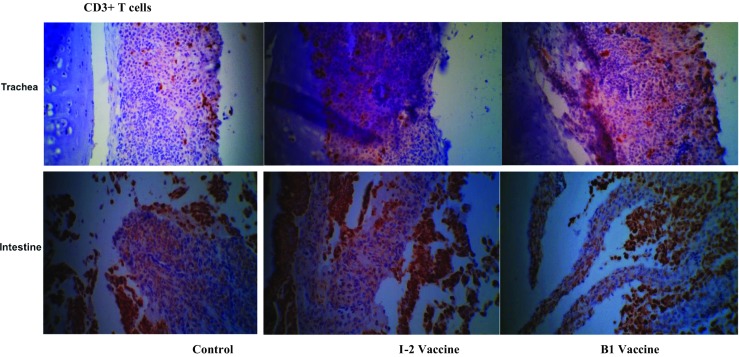
Immunohistochemistry representative images of tracheal and duodenal samples of the I-2 thermostable and B1 vaccinated groups and the unvaccinated control group. Stained with CD3+ monoclonal antibody and sectioned 3 days post-booster vaccine administration. ×400 magnification

We have used CD3+, CD4+, and CD8+ homolog antibodies to describe lymphocyte distribution changes in the infection period according to the vaccine type and Newcastle challenge. The total number of T lymphocytes (CD3+) and studied subsets (CD4+ and CD8+) was differently distributed in the studied tissues (Figs. 10, 11, and 12).

Total CD3+, CD4+, and CD8+ numbers of the vaccinated group’s trachea and intestine samples (I-2 and B1) were significantly ascending compared with the negative control group, and therefore, the changes were significant, too (*P* ˂ 0.001). The numbers of CD3+ cells were not significant in various days and vaccinated groups (B1 and I-2) in the tracheal but in the intestinal samples. The level of CD3+ cell changes was significant in the intestinal and tracheal samples of vaccinated groups (B1 and I-2) compared with the negative control group.

On the whole, a significant difference was not noticed between the number of tracheal CD4+ cells of the vaccinated groups (I-2 and B1) but between them and the negative control group. The number of tracheal CD4+ of the vaccinated groups (I-2 and B1) was only significantly different 14 days after the administration.

The total difference between number of CD4+ cells of the vaccinated groups (I-2 and B1) was significant. It was also significant compared with the negative control group. The number of CD4+ cells was increased more 7 days after administering the booster of I-2 group making the difference more significant.

The numbers of CD8+ cells of the tracheal and intestinal samples of the vaccinated groups (I-2 and B1) were not significantly different. However, the difference was significant compared with the negative control group. The difference between the numbers of intratracheal CD8+ cells was significant 3 and 14 days after the administration. The level of CD8+ was higher in B1 group 3 days after administering the vaccine while it reversed 14 days after.

Seven days after administering the primary vaccine, the numbers of CD3+, CD4+, and CD8+ cells were increased making the difference with the number of negative control group significant. The numbers of CD4+ and CD8+ cells were peaked in both tissues 7 days after administering the booster vaccine.

The number of tracheal and intestinal CD4+ cells was two or three times significantly higher than the negative control group 7 days after the primary vaccine administration. The trend was still ascending up to 7 days after the booster vaccine administration making the difference three to four times higher than the negative control group.

The number of tracheal and intestinal CD8+ cells was three or four times significantly higher than the negative control group 7 days after the primary vaccine administration. The trend was still ascending up to 7 days after the booster vaccine administration making the difference seven to eight times higher than the negative control group.

Most changes in increasing trend of CD3+, CD4+, and CD8+ numbers were occurred in two first weeks after the infection. The trend was slightly decreasing 3 weeks post-infection and then again ascending up to 4 weeks post-infection or a week after the challenge.

The numbers of CD3+, CD4+, and CD8+ cells in the tracheal and intestinal samples of I-2 and B1 vaccinated chicks, which were then challenged with acute Newcastle virus, were significantly different than the negative control group 7 days post-challenge. However, the difference was not significant between two vaccinated groups.

The ratio of CD4+/CD8+ changes of intestinal and tracheal samples was not significantly different. In other words, there was no significant difference between the averages of the ratios in various time points. Both I-2 and B1 vaccinated groups were not significantly different with each other but with the negative control group.

The ratio of CD4+/CD8+ was 2 to 1 in the healthy chicks (negative control group), while it was 1 to 1 after administering the vaccines. The numbers of CD8+ cells were higher than the CD4+ cells after the vaccination but not significantly. Totally, both numbers of CD4+ and CD8+ cells were increasing, but the trend was more obvious in CD8+ cells.

## Discussion and conclusion

To improve the vaccination strategies in the field and to evaluate the vaccines, it is necessary to increase our knowledge about immunostimulatory conditions by the vaccine and the challenge virus (Noraup et al. 2011). Several studies have been conducted to evaluate the immune response to infection with NDV. Some of them suggested that innate immunity can play an important role against NDV infection (Bing-guo et al. 2011; Rauw et al. 2009). Recent studies also confirmed detection of CMI reaction to NDV, shortly after vaccination with a live vaccine (Reynolds and Maraqa 2000).

Immunohistochemistry is an accurate and specific immunoassay technique that could indicate the tissue distribution of the selected agents in tissue sections. Several studies have been conducted to evaluate the effects of viruses and various vaccines such as HVT, MDV, IBDV, and so forth on the infiltration of T cell subtypes (CD4+ and CD8+) using IHC method (Kamran et al. 2010; Silke and Christine 2005; Takao et al. 1999). Also, some limited studies have been conducted to evaluate the distribution of T cell subtypes (CD4+ and CD8+) following the vaccination and challenge with Newcastle disease virus (Xiaofei et al. 2007; Awad et al. 2014; Russell et al. 1997). On the other hand, interferon-γ ELISA test indicated a high potential in measuring CMI role in protecting the chickens against the poultry infectious diseases in the future and studying the role of interferon-γ in various immune mechanisms of the chickens (Lambrecht et al. 2004). Interferon-γ (interferon type II) is secreted by a few immune cells (including T helpers, cytotoxic T cells, and natural killer cells) with antiviral and immunostimulatory effects (Staeheli et al. 2001). Overall, this type of immune response could affect on the type of cellular immunity involved in NDV clearance (Agrawal and Reynolds 1991; Reynolds and Maraqa 2000; Seal et al. 2000). However, limited data is available regarding the functions of interferon-γ in the avian immune system, and the knowledge regarding this interferon is not well developed in various avian diseases including the Newcastle disease (Susta et al. 2013).

In a study on the increased of γ interferon levels in the chicken’s splenocytes infected with California acute (CA) strain comparing with a lentogenic Newcastle virus strain, the increment of interferon-γ level in the sera of acute strain-infected chicks was noticed 2 and 3 days PI (Rue et al. 2011). In addition, it was indicated that very virulent Newcastle disease virus (velogenic viscerotropic strain, ZJ1) induced high level of interferon-γ production in the backyard poultry, just prior to death, while the vaccine strain did not have similar effect (Cornax et al. 2012). Cellular immunity response was evaluated by measuring interferon-γ with ELISA method after splenocyte stimulation by mitogens and antigen induction by vaccinating with inactive and live LaSota vaccine. Many vaccinated chicks produced interferon-γ 2 to 4 weeks after stimulation of vaccinating with a live vaccine. In case of the inactive vaccine, half of the chicks responded significantly 4 weeks after the vaccination. This indicated that the sensitivity of ELISA in measuring interferon-γ production by T cells in the response to mitogens and specific antigen induction in in vitro conditions was sufficient (Lambrecht et al. 2004).

The effects of three antibiotics with vaccinating with LaSota live and inactive vaccination in the cellular immunity response were assessed in 20-week SPF chickens by measuring interferon-γ in the cell culture using ELISA. Five days after cell culture inoculation, the group received antibiotics and the vaccine simultaneously showed a significant increment in interferon-γ production compared with the control group and the group that received antibiotics only (Khalifeh et al. 2009). The importance of interferon-γ was indicated using IFN-γ and levamisole as the adjuvant in Newcastle DNA vaccine. Selecting this cytokine as the adjuvant would enhance the protection of the chicks against the acute Newcastle disease virus challenge (Yin et al. 2006). Evaluating the cellular immunity in the birds is cumbersome and hence is not a common practice in this field.

As mentioned above, measuring interferon-γ as a cytokine after the stimulation could be an appropriate evaluation method for the cellular immunity. Interferon-γ changes were significant in the period of this study (*p* < 0.001), and the levels of its titer were increasing. There was no significant difference between two groups received I-2 and B1 vaccines. However, a significance difference was noticed between I-2 group with negative control and B1 groups. Significant different serum levels of these parameters were also noticed in different days (*p* < 0.001). The level of interferon-γ started to increase 3 days after the vaccination and peaked 6 days post-challenge in the vaccinated groups. Cellular immunity has an important role in many immune responses against the viruses with various mechanisms such as cytokine production (Kaiser and Staheli 2008). It was indicated in a study that interferon-γ could decrease the effects of Newcastle virus if only its level is high at the beginning of the infection. It was suggested that interferon-γ might induce a delayed and insufficient response against the virus replication. The virus load and its replication might be decreased with its direct anti-viral effect, changes in innate immune response by its immunostimultory effects, or a combination of both (Peiris et al. 2009, 2010).

### Evaluating T cell (CD4+, CD8+) and whole lymphocyte changes using immunohistochemistry

The aim of this part of the study was to detect the infiltrated lymphocytes into the tissues and the immunologic mechanism of the chicks against the infection. Lymphocytes are usually found as mucosal-associated lymphoid tissue in the mucus of the fowl (Bar-Shira et al. 2003). It is well known that cellular immunity protects the body against the viral pathogens with (1) inducing cytotoxic activity, (2) detecting target antigen related to major histocompatibility complex (MHC), and (3) producing lymphokines such as interferons and interleukin 2 and tumor necrosis factor-β. Various activities of mediator cells could be detected and differentiated by cellular surface antigens. CD4+ and CD8+ are used to detect T helper and T cytotoxic cells, respectively, while CD3+ is used as a common antigen with T cells (Vainio and Lassila 1989).

In a study, the effects of combining two adjuvants (cMIA I and cMIA II) with Newcastle disease virus on the mucosal and systemic immune responses were studied. It was indicated that local changes of CD3+ cells in duodenum and jejunal of all groups were ascending after the vaccination. The numbers of CD3+ lymphocytes in the groups receiving adjuvant and Newcastle disease vaccine were higher than the negative control group (Xiaofei et al. 2007). The interactions between NDV live vaccine virus (VG/GA strain), metapneumovirus, and infectious bronchitis using the immunohistochemistry method were studied in young SPF chicks. Twenty-one days after the vaccination, significant expression of tracheal CD4+ and CD8+ lymphocytes was noticed in the vaccinated group compared with the control group. The levels of CD4+ and CD8+ were approximately 15 and 10 times higher than the negative control group (Awad et al. 2014).

In a study, the changes of B and T cells were studied in the areas of virus replication after the vaccination using immunohistochemistry method. To study the roles of B and T cells in scavenging the virus, cyclophosphamide (CY) and cyclosporine A (cyclosporine) were used in B and T cell suppression. NDV vaccination results in two or three times increment in the numbers of CD3+ and CD4+ cells compared with the non-treated chicks. CD8+ cells were increased six times. In chicks treated with cyclophosphamide, all T cell subtypes were increased two to four times after the vaccination. In chicks treated with cyclosporine, the numbers of CD4+ cells were increased three to four times after the vaccination achieved to the levels of healthy and non-vaccinated chicks. CD8+ cells were also increased 10 times. This study was to indicate the details of local antiviral response of T cells in newly hatched chicks vaccinated with B1 Hitchner strain. The increment of T cells in the first sampling on 3 days after the vaccination was obvious. All T cell subtypes were increased as a result of vaccination at least two times. Cd8+ was increased 6 and 11 times in non-treated and cyclosporine-treated birds, respectively. On the other hand, CD4+ cells were also increased two and three times in non-treated chicks (Russell et al. 1997). The ratio of CD8+ cells to CD4+ cells was 1:2 in non-vaccinated chicks, and then, it was changed to 1:1 after the vaccination. As indicated, the cytotoxic response of CD8+ lymphocytes is necessary to primarily control, treat, and reactivate viral infections (Farrel and Davis-Poynter 1998). Takao et al. (1999) studied the lymphocyte subtypes in the trachea of the chickens inoculated with infectious bronchitis virus and concluded that the chicken’s immune system might use the cytotoxic effects of CD8+ lymphocytes to inactivate the virus in the primary stages of the infection and then system relies on the humoral immunity to control the viral infection. Songserm et al. (2000) also observed the increment of CD8+ in the intestine of the chickens inoculated with malabsorption syndrome homogenate. In addition to CD4+ and CD8+ cells, natural killer cells might play important roles in protecting against the digestive system pathogens.

Although the source of interferon-γ was not evaluated in the current study, this cytokine could be expressed by NK, CD4+, and CD8+ cells (Parvizi et al. 2009; Garcia-Camacho et al. 2003). Also, expanding of T cell subtypes in the trachea and the intestine was observed following the infection or vaccination with Newcastle virus. Interferon-γ might activate CD8+ cells in addition to direct antiviral effects to scavenge the infected cells (Whitmire et al. 2005). In our study, the number of CD8+ cells was significantly increased in the vaccinated group compared with the non-vaccinated non-challenged group. Although CD4+ cells play less important roles in scavenging the virus-infected cells directly, these cells might be effective in CD8+ maturation and differentiation into antigen-specific CTLs. Omar and Schat indicated that CD4+-specific CTLs are not important in destroying the cells expressing the viral antigens (Omar and Schat 1997).

Finally, it was concluded that administering thermostable I-2 and B1 Newcastle vaccines could protect the chickens against the acute Newcastle virus. Our findings indicated the significant increments of CD4+ and CD8+ expression in the intestinal and tracheal tissues together with ascending interferon-γ level in the sera of vaccinated groups compared with the non-vaccinated one. Finding no significant difference between the vaccinated groups indicated that the ability of both vaccines in producing CD4+ and CD8+ in tracheal and intestinal tissues and also the level of serum interferon-γ are similar.
